# Quantifying Intervention Effects in Single-Case Research: A 25-Year Review

**DOI:** 10.3390/bs16040507

**Published:** 2026-03-28

**Authors:** Serife Balikci, Emrah Gulboy

**Affiliations:** 1 Early Childhood Education/Teacher Preparation Programs, Piedmont Community College, Roxboro, NC 27574, USA; 2 Carsamba District Directorate of National Education, Samsun 55500, Türkiye; 3 Department of Special Education, School of Education, Ondokuz Mayis University, Samsun 55270, Türkiye; emrah.gulboy@omu.edu.tr

**Keywords:** single-case research design, effect size reporting, special education, systematic review, PND, Tau-*U*, evidence-based practice

## Abstract

The use of effect size estimates to complement visual analysis has become increasingly emphasized in single-case research design (SCRD) studies in special education. This review examined trends in SCRD publication and ES reporting practices across three major special education journals (i.e., Journal of Special Education, Exceptional Children, and Remedial and Special Education) from 2001 to 2025. A total of 1969 articles were screened, yielding 194 SCRD intervention studies and 124 SCRD systematic reviews/meta-analyses. Results indicated a sustained increase in the publication of SCRD studies over time, accompanied by a marked rise in ES reporting. Overall, 42.27% (*n* = 82) of SCRD intervention studies and 55.65% (*n* = 69) of SCRD systematic reviews reported at least one effect size estimate, with the highest rates observed in the most recent publication period (2001–2025). Across both intervention studies and systematic reviews, Percentage of Nonoverlapping Data and Tau-*U* were the most frequently reported ES metrics, although reliance on Percentage of Nonoverlapping Data declined over time while use of Tau-*U* increased. Findings highlight evolving effect size reporting practices and have implications for evidence synthesis and methodological standards in special education research.

## 1. Introduction

The movement toward identifying evidence-based practices (EBPs) in special education gained significant momentum following the enactment of the No Child Left Behind (NCLB) Act in 2001 and the subsequent reauthorization of the Individuals with Disabilities Education Improvement Act in 2004. More recently, the Every Student Succeeds Act, which replaced NCLB, has further underscored the importance of educational professionals implementing EBPs in classrooms when teaching students with and without disabilities. Collectively, these federal education laws emphasize the use of academic, social, emotional, and behavioral strategies that are firmly rooted in scientifically based research to the greatest extent feasible (Cook & Cook, 2011). In response to these legislative mandates and the growing demand for rigorous, research-based decision making in education, the U.S. Department of Education established the What Works Clearinghouse (WWC) as a centralized mechanism to evaluate and disseminate evidence on the effectiveness of educational interventions. The WWC supports the implementation of evidence-based practices (EBPs) by conducting systematic reviews of research using transparent and standardized criteria, thereby providing educators, policymakers, and researchers with trustworthy information about practices supported by strong empirical evidence (WWC, 2020). By translating complex research findings into accessible guidance, the WWC helps bridge the gap between federal policy expectations and classroom practice.

Consistent with this policy-driven emphasis, the extant literature confirms that EBPs yield numerous benefits for both students and teachers. Specifically, EBPs are more likely to produce favorable student outcomes, promote efficient allocation of instructional resources such as time and materials, and enhance professional accountability by encouraging educators to base instructional decisions on the most current and credible empirical evidence available (Cook et al., 2011, 2020).

## 2. The Role of Effect Sizes and Quality Indicators in Establishing EBPs

The federal law’s requirements pertaining to the utilization of EBPs, coupled with the recognized benefits of implementing such practices as documented in the existing literature, have significantly expedited the identification of EBPs (Cook & Odom, 2013). In light of this imperative, several established organizations, including but not limited to the Center for Research and Reform in Education, the National Autism Center, the National Center on Response to Intervention, the National Professional Development Center on Autism Spectrum Disorder, the National Secondary Transition Technical Assistance Center, the Promising Practices Network, and the WWC, have actively engaged in the task of identifying EBPs in special education (Balıkcı et al., 2022; Yucesoy-Ozkan et al., 2020). These entities and researchers have frequently employed systematic reviews, meta-analyses, or comprehensive syntheses of individual research studies focused on specific practices or interventions to establish the evidentiary foundation for such practices or interventions (Jamshidi et al., 2022). Moreover, they have developed training modules and instructional materials to facilitate educators’ use of EBPs in classrooms.

Initially, systematic reviews and meta-analyses often excluded studies that employed single-case research designs (SCRD). This exclusion was not only due to reliance on visual analysis but also to methodological differences from traditional group-experimental research, such as randomized controlled trials, which are typically regarded as the “gold standard” in research methodology. Critics pointed to issues such as the lack of randomization of participants and distinct generalization logic in SCRD compared to group designs (Brossart et al., 2006; Wolery, 2013). However, implementing group-experimental methods in special education poses significant logistical challenges, such as recruiting large sample sizes given the low incidence of certain disabilities (Maggin et al., 2021; Mitchell & Karchmer, 2004). In contrast, SCRD offers distinct advantages, including the ability to monitor intervention effectiveness continuously allows for immediate adjustments, and it eliminates the ethical concern of withholding intervention from a control group, which is often deemed unacceptable for children with severe academic delays (Shadish et al., 2015). While visual analysis remains a fundamental tool for identifying functional relations in SCRD (Ledford et al., 2018b), the growing emphasis on identifying EBPs has led to the increased use of effect size (ES) estimates as a supplement to facilitate the aggregation of SCRD findings (Rakap, 2015).

While proponents of visual analysis argue for its exclusive usage (e.g., Wolery et al., 2010), others have advocated for the incorporation of ES estimates to augment visual analysis and facilitate the meta-analysis of studies employing SCRD (e.g., Kratochwill et al., 2010; WWC, 2020). Visual analysis provides valuable insights into intervention effectiveness by examining the temporal relationship between interventions and outcomes. On the other hand, effect size metrics play a complementary role by summarizing the magnitude of intervention effects within and across studies. These metrics offer a standardized approach for quantifying intervention impacts, enabling researchers to compare and synthesize findings across studies (Yucesoy-Ozkan et al., 2020). Importantly, effect size metrics are not intended to replace visual analysis but to supplement it once a functional relation has been established (Moeyaert et al., 2018). This shift reflects a growing recognition of the potential benefits associated with leveraging ES estimates in identifying EBPs.

Reporting ES is a common practice among researchers undertaking group experimental studies (Cook et al., 2018; Durlak, 2009; Kraft, 2020; Thompson, 2007). The American Psychological Association and the American Educational Research Association explicitly require the reporting of ES when quantitative research methods are used (Peng et al., 2013; Schäfer & Schwarz, 2019). Moreover, prominent special education journals, as evidenced by impact factors, such as the Journal of Special Education, Exceptional Children, and Remedial and Special Education, now either require or recommend that researchers report ES when appropriate (Appelbaum et al., 2018).

Considering norms established by researchers using group experimental designs, as well as the current emphasis on identifying EBPs in special education, Kratochwill et al. (2010) recommended the use of ES estimates to supplement visual analysis in the pilot version of the Single-Case Design Technical Documentation developed as part of the WWC’s effort to expand the pool of scientific evidence available for review. More recently, other researchers have further discussed the importance of reporting ES estimates when presenting results of SCRD studies (e.g., Maggin et al., 2021; Parker et al., 2014; Pustejovsky & Ferron, 2017; Rakap, 2015; Vannest et al., 2018). Moreover, authors of the latest version of the WWC Standards Handbook, Version 4.1 (WWC, 2020), recommend the use of design-comparable effect sizes (D-CES) to estimate treatment effects in SCRD studies. D-CES estimates align with the metrics (e.g., Cohen’s d) used to calculate ES in group- or quasi-experimental studies (Kratochwill et al., 2021). This shift in standards allows SCRD studies to be considered in evidence reviews conducted by WWC-affiliated reviewers and personnel (Maggin et al., 2021).

Several initiatives have also been undertaken by professional organizations to enhance the methodological quality of SCRD studies, including the development of quality standards (Cook et al., 2015; Horner et al., 2005; Ledford et al., 2023), recommendations (Rodgers et al., 2018; Wolery et al., 2011), and checklists and rubrics (Maggin et al., 2014). These endeavors have yielded more rigorous and well-designed SCRD studies, which hold substantial potential for contributing to the knowledge base on effective practices for supporting students with disabilities (Vannest et al., 2018). To ensure the inclusion of these high-quality SCRD studies in EBP syntheses, systematic reviews, and meta-analyses, the calculation of ES estimates becomes imperative for integrating findings across multiple studies in which researchers investigated special education interventions and practices.

## 3. Approaches for Determining Effect Sizes in SCRD

SCRD researchers have identified a wide range of approaches for computing ES estimates and determining treatment effects. These metrics are broadly categorized into three groups: overlap metrics, mean-based metrics, and regression-based approaches (Moeyaert et al., 2018; Rakap et al., 2014). The overlap metrics include, but are not limited to, the Percentage of Nonoverlapping Data (PND), Percentage of Data Exceeding the Median (PEM), Percentage of All Nonoverlapping Data (PAND), Nonoverlap of All Pairs (NAP), Improvement Rate Difference (IRD), Percentage of Data Exceeding the Median Trend (PEM-T), Tau, Tau Nonoverlap, Tau-*U*, and Tau-C. The mean-based metrics include the No-Assumptions Effect Size (NAES), Swanson’s procedure, log response ratio (LRR), SMD, between-case SMD, d-statistic, and d-Hedges–Pustejovsky–Shadish (DHPS). The regression-based approaches include hierarchical linear modeling and the Pustejovsky–Hedges–Shadish d (Moeyaert et al., 2018; Rakap et al., 2020; Vannest et al., 2018; Yucesoy-Ozkan et al., 2020).

Each of these approaches is associated with distinct advantages and limitations, and the data characteristics commonly observed in SCRD studies present unique challenges for estimating treatment effects using these methods (Moeyaert et al., 2018; Rakap, 2015). Consequently, SCRD researchers have not yet reached consensus regarding the use of a single ES index for calculating treatment effects (Vannest et al., 2018; Wolery et al., 2010). Additionally, variability in SCRD data characteristics—such as trend, autocorrelation, number of data points, and stability—necessitates the use of different ES metrics across studies (Yucesoy-Ozkan et al., 2020). Therefore, it is recommended that researchers carefully select ES metrics based on the specific data characteristics of each study and, when appropriate, report results from multiple calculations to facilitate comparison and interpretation (Vannest et al., 2018; Yucesoy-Ozkan et al., 2020).

## 4. Previous SCRD Reviews

Researchers have examined various aspects of SCRD studies, including ES reporting practices across different journals and fields. Beretvas and Chung (2008) conducted a review of 25 SCRD meta-analyses published between 1985 and 2005 across multiple disciplines. They found that most meta-analysts relied on PND (48%), percentage of zero data (48%), and SMD (28%) to calculate ES estimates. Of the 25 meta-analyses, seven (28%) used multiple ES metrics to describe their results. Farmer et al. (2010) examined 39 SCRD meta-analyses published between 1999 and 2009 across various social science disciplines and similarly found that PND was the most frequently used ES metric. Maggin et al. (2011) reviewed 68 SCRD meta-analyses published between 1985 and 2009 and reported a steady increase in SCRD meta-analyses during that period, with PND (55%) being the most frequently applied ES metric, followed by SMD (19%). Among these studies, 54 (80%) reported a single ES metric, 11 (16%) reported two metrics, two (3%) reported three metrics, and one (1.5%) reported four metrics.

More recently, Radley et al. (2020) examined publication trends in school psychology research employing SCRDs and found that 24.5% of SCRD studies published between 1968 and 2015 reported an ES estimate. Across these studies, nine different ES metrics were identified, with the No-Assumption Index being the most frequently used (26%), followed by PND (22%) and Cohen’s d (15%). Jamshidi et al. (2022) reviewed 178 SCRD meta-analyses published between 1985 and 2015 and documented a substantial increase in the number of SCRD meta-analyses over time. The most commonly used ES metric was PND (61%), followed by SMD (20%) and IRD (16%), whereas Tau-*U* (6%) was among the least frequently used metrics.

Although the use of ES estimates to supplement visual analysis in SCRD has been increasingly recommended (Maggin et al., 2021), limited empirical evidence exists regarding the specific ES reporting practices in published special education research employing SCRDs. Consequently, it remains unclear how frequently SCRD researchers report ES estimates and which metrics are used across journals and over time. Understanding ES reporting practices in SCRD is important for several reasons. First, it provides insight into the extent to which SCRD researchers report ES estimates in special education research and helps characterize current reporting norms. Second, examining the range of ES metrics used in SCRD allows researchers to evaluate the diversity, applicability, and consistency of different approaches. Finally, identifying gaps in ES reporting practices highlights areas in need of methodological guidance. The limited understanding of ES reporting practices in SCRD underscores the need for systematic investigation. Addressing this gap can inform the development of data-informed guidelines, recommendations, and standards for ES reporting in SCRD, ultimately strengthening the methodological rigor and interpretability of research in special education.

The purpose of this review is to investigate the reporting trends of ES by special education researchers employing SCRD in published intervention studies as well as SCRD systematic reviews and meta-analyses. The study focuses on published articles across five publication periods (2001–2005, 2006–2010, 2011–2015, 2016–2020, and 2021–2025) in three major special education journals: the Journal of Special Education (JSE), Exceptional Children (EC), and Remedial and Special Education (RASE). In this review, we addressed the following research questions:

1. What is the prevalence of SCRD intervention studies and SCRD systematic reviews/meta-analyses published in the JSE, EC, and RASE, and how has the publication of these SCRD article types changed over time?
2. What is the prevalence of SCRD intervention studies and SCRD systematic reviews/meta-analyses published in the JSE, EC, and RASE that include at least one reported ES metric to complement visual analysis, and how has the practice of reporting ES changed over time?
3. Which ES metrics are prevalent for reporting ES in SCRD intervention studies and SCRD systematic reviews/meta-analyses published in the JSE, EC, and RASE, and how has the selection of ES metrics changed across publication periods?

## 5. Method

We conducted this review based on SCRD studies published in three prominent journals: the Journal of Special Education (JSE), Exceptional Children (EC), and Remedial and Special Education (RASE), covering the period from 2001 through the most recent publication years included in the review (i.e., 2001–2025). We selected these journals because of their high impact factors and influence in the field of special education (i.e., JSE = 2.2, EC = 4.3, RASE = 3.4). We established the range of years to encompass the decade preceding the publication of the WWC Single-Case Design Technical Documentation (Kratochwill et al., 2010), which represented one of the earliest formal recommendations for reporting ES estimates to supplement visual analysis in SCRD, as well as subsequent years reflecting evolving reporting practices. At the time of the review, EC submission guidelines required researchers to report ES for SCRD studies, JSE guidelines recommended ES use when appropriate, and RASE author guidelines did not explicitly address ES use.

In addition to their high impact factors, these journals were selected because they are widely recognized as leading outlets for empirical research in special education and have historically published a substantial proportion of SCRD studies. As flagship journals of major professional organizations and the broader special education research community, they often influence methodological expectations and reporting standards within the field. Consequently, examining SCRD publication and effect size reporting practices within these journals provides insight into how reporting norms may be evolving in prominent and widely cited publication venues in special education. Although reporting practices in other journals may differ, focusing on these influential outlets allowed the present review to capture trends in journals that are likely to shape broader research and publication practices in the field.

### 5.1. Article Search and Selection Procedures

In the current study, we included published articles in which researchers reported the results of individual SCRD intervention studies, as well as systematic reviews, syntheses, and meta-analyses of SCRD studies (hereafter referred to as systematic reviews). To gather relevant published articles, we conducted an electronic search of JSE, EC, and RASE through the library systems of the University of North Carolina Greensboro (USA) for all issues published between 2001 and 2025. We downloaded all publications, regardless of research method or article type, for further screening. Subsequently, we recorded the number of publications per year per journal on an author-created Excel spreadsheet. Once all articles were retrieved, we screened their titles, abstracts, and, when necessary, full texts to determine whether they met the inclusion and exclusion criteria.

To be included in this review, published articles were required to report results from an SCRD intervention study or a systematic review that synthesized SCRD studies. We included SCRD articles regardless of the specific single-case design employed (e.g., alternating treatment, multiple baseline), without restrictions related to methodological quality or experimental rigor. Studies employing other research designs (e.g., quantitative group designs, mixed methods, qualitative inquiries) were excluded. Systematic reviews were included when they synthesized multiple SCRD studies or a combination of SCRD and group-design studies. Reviews based solely on qualitative studies, quantitative group-design studies, or methodological or conceptual articles were excluded. Importantly, neither SCRD intervention studies nor systematic reviews were required to report an ES estimate for inclusion at this stage. During the initial screening, articles were categorized dichotomously as either reporting SCRD intervention results or representing a systematic review that included SCRD studies.

**Figure 1:**
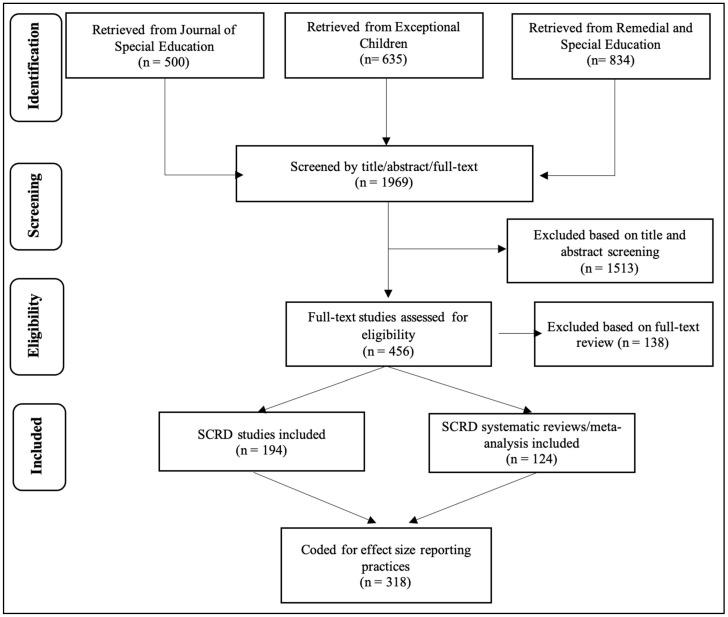
Flowchart of Search Procedure Used to Identify Studies Included in this Review. Note. SCRD = Single-Case Research Design.

As illustrated in Figure 1, the article selection process yielded a total of 1969 articles across the three journals. After screening titles and abstracts, 1513 articles were excluded because they did not report SCRD intervention results or systematic reviews. The remaining 456 articles underwent full-text review, resulting in the exclusion of an additional 138 articles for the same reasons. This process yielded a total of 318 articles for inclusion, consisting of 194 SCRD intervention studies and 124 systematic reviews.

To ensure the reliability of the screening process, the second author independently screened all 1969 articles. Inter-rater agreement (IRA) was calculated by dividing the total number of agreements by the total number of agreements and disagreements (Ledford et al., 2018a). The IRA for screening and selecting eligible studies was 99.5%, indicating a high level of agreement between reviewers. Disagreements were resolved through discussion until consensus was reached. The first author, a doctoral-level researcher in special education, possessed expertise in conducting systematic reviews of SCRD studies and research focused on ES methods in SCRD. Similarly, the second author held a doctoral degree in special education and had extensive experience conducting systematic reviews and studies examining ES methodologies in SCRD research.

### 5.2. Coding Procedures

Following article selection, the first author reviewed the Method and Results sections of the 318 included articles (i.e., 194 SCRD intervention studies and 124 systematic reviews) to determine whether ES estimates were reported to complement visual analysis. For each article, the author(s) name, journal, and publication year were recorded in an author-created Excel spreadsheet. If an article did not report an ES estimate, no additional coding was conducted. When an article reported one or more ES estimates, we coded the specific ES metric(s) used, following the classification framework described by Radley et al. (2020). For analytic purposes, articles that did not report an ES estimate were retained for prevalence analyses but excluded from analyses examining ES metric use. As a result, articles reporting SCRD intervention studies or systematic reviews without ES estimates were excluded from metric-specific analyses (*n* = 167). This yielded a final analytic dataset of 151 articles that reported at least one ES estimate to supplement visual analysis.

To ensure coding reliability, the second author independently coded a randomly selected 40% of the articles across each journal (Ledford et al., 2018a). Inter-rater reliability for ES coding was 96%, indicating a high level of agreement. Any discrepancies were resolved through discussion until consensus was reached.

### 5.3. Data Analysis

We used descriptive statistics to analyze the data, as the primary purpose of the study was to describe patterns and trends in SCRD publication and ES reporting practices rather than to draw inferential conclusions. Specifically, descriptive analyses were conducted to determine the frequency and percentage of (a) SCRD intervention studies and systematic reviews published in each journal and across journals, and (b) SCRD intervention studies and systematic reviews that reported at least one ES estimate. Additionally, descriptive analyses were used to identify the frequency and percentage of specific ES metrics reported across studies.

To examine temporal trends, articles were grouped into five publication periods: 2001–2005, 2006–2010, 2011–2015, 2016–2020, and 2021–2025. These periods were selected to correspond with the organization of the data tables and to reflect meaningful phases in the evolution of SCRD quality standards and ES reporting guidance. The first period (2001–2005) represented years preceding the publication of SCRD quality standards (Horner et al., 2005). The second period (2006–2010) captured the years following these standards and leading up to the release of the WWC Single-Case Design Technical Documentation (Kratochwill et al., 2010). The third period (2011–2015) reflected the years immediately following the WWC technical documentation, whereas the fourth (2016–2020) and fifth (2021–2025) periods represented more recent years.

## 6. Results

### 6.1. Prevalence of SCRD Intervention Studies and Systematic Reviews

We identified a total of 1969 articles published across the three special education journals between 2001 and 2025. Following the screening of titles and abstracts, 1513 articles were excluded. The remaining 456 articles underwent full-text review, resulting in a final inclusion of 318 articles. Of these articles, 194 (9.85%) reported results from SCRD intervention studies, and 124 (6.30%) reported results from systematic reviews or meta-analyses that included SCRD studies (see Table 1 and Table 2). When examined by journal, the Journal of Special Education (JSE) published 500 articles, of which 82 (16.4%) were SCRD intervention studies and 22 (4.40%) were SCRD systematic reviews. Exceptional Children (EC) published 635 articles, including 46 (7.24%) SCRD intervention studies and 29 (4.57%) SCRD systematic reviews. Remedial and Special Education (RASE) published 834 articles, with 66 (7.91%) SCRD intervention studies and 73 (8.75%) SCRD systematic reviews.

**Figure 2:**
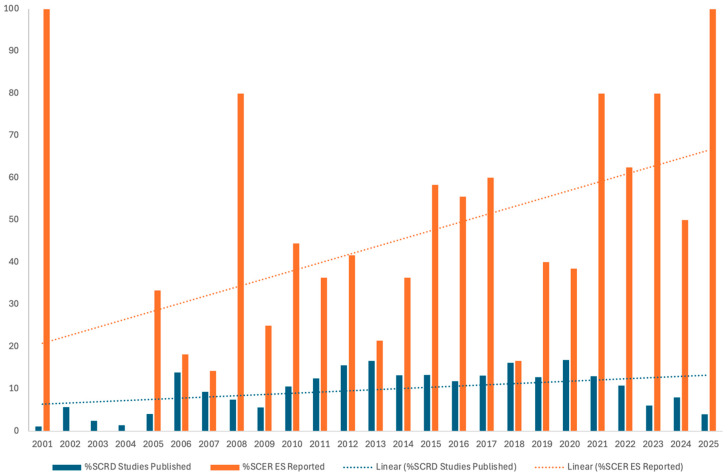
Percentage of SCRD Studies Published and Reported Effect Sizes. Note. SCRD = Single-Case Research Design.

**Figure 3:**
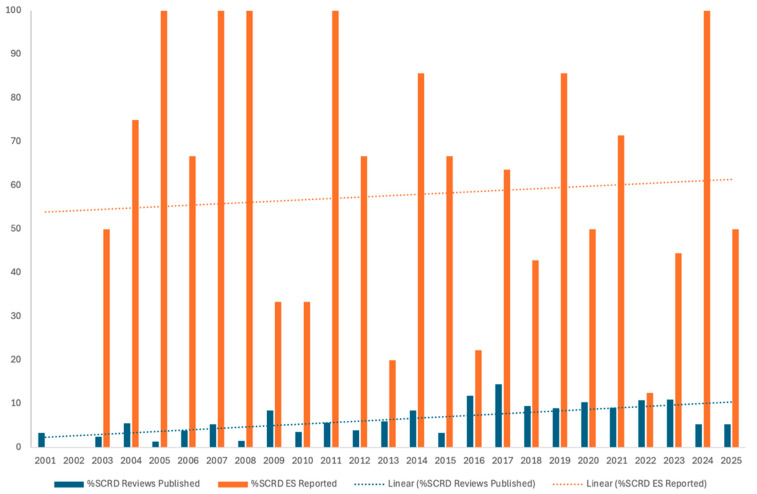
Percentage of SCRD Reviews/Meta-Analysis Published and Reported Effect Sizes. Note. SCRD = Single-Case Research Design.

As shown in Figure 2 and Table 1, the percentage of SCRD intervention studies published across the three journals increased over time. Specifically, SCRD intervention studies accounted for 2.96% of all published articles between 2001–2005, increased to 9.55% between 2006–2010, 14.22% between 2011–2015, and 14.17% between 2016–2020, before declining slightly to 8.36% during 2021–2025. Across all five periods, SCRD intervention studies represented an average of 9.85% of published articles. Journal-level trends showed variability. In JSE, the percentage of SCRD intervention studies increased from 6.25% (2001–2005) to 23.36% (2016–2020) before decreasing to 14.15% (2021–2025). EC demonstrated a gradual increase from 2.90% to 10.14% between 2001–2005 and 2011–2015, followed by relatively stable rates through 2025. RASE showed a consistent increase from 1.16% (2001–2005) to 13.19% (2011–2015), with lower representation in the most recent period (4.85%). A similar pattern was observed for systematic reviews and meta-analyses (Table 2 and Figure 3). Across journals, the percentage of SCRD systematic reviews increased from 2.46% (2001–2005) to 11.02% (2016–2020) and remained at 8.36% during 2021–2025. Overall, SCRD systematic reviews represented 6.30% of all published articles.

### 6.2. Prevalence of Studies Reporting Effect Sizes

Across the three journals, 82 of the 194 SCRD intervention studies (42.27%) reported at least one effect size (ES) estimate to complement visual analysis (Table 1). The number and proportion of SCRD intervention studies reporting ES estimates increased markedly across publication periods. Specifically, 2 of 12 studies (16.67%) reported ES estimates between 2001–2005, increasing to 12 of 36 studies (33.33%) during 2006–2010, 23 of 60 studies (38.33%) during 2011–2015, and 22 of 54 studies (40.74%) during 2016–2020. The highest rate of ES reporting occurred during 2021–2025, when 23 of 32 SCRD intervention studies (71.88%) included at least one ES estimate.

Journal-level analyses revealed differences in both the overall prevalence and temporal patterns of ES reporting. In Exceptional Children (EC), 22 of 46 SCRD intervention studies (47.83%) reported ES estimates, representing the highest overall proportion among the three journals. ES reporting in EC increased from 1 of 4 studies (25.00%) in 2001–2005 to 7 of 9 studies (77.78%) in 2021–2025, with notable variability across intermediate periods (e.g., 28.57% in 2011–2015 and 44.44% in 2016–2020). In Remedial and Special Education (RASE), 27 of 66 SCRD intervention studies (40.91%) reported ES estimates. Although only 1 of 2 studies (50.00%) reported ES estimates during 2001–2005, ES reporting declined to 1 of 12 studies (8.33%) during 2006–2010 before increasing steadily across subsequent periods. By 2021–2025, 7 of 8 SCRD intervention studies (87.50%) in RASE reported ES estimates, representing the highest journal-specific rate observed in the dataset. In the Journal of Special Education (JSE), 33 of 82 SCRD intervention studies (40.24%) included ES estimates. No SCRD intervention studies published in JSE during 2001–2005 reported ES estimates (0 of 6 studies; 0.00%). ES reporting increased in later periods, with 5 of 14 studies (35.71%) during 2006–2010, 9 of 22 studies (40.91%) during 2011–2015, 10 of 25 studies (40.00%) during 2016–2020, and 9 of 15 studies (60.00%) during 2021–2025 reporting ES estimates.

Among the 124 SCRD systematic reviews, 69 reviews (55.65%) reported at least one ES estimate (Table 2 and Figure 3). Across publication periods, ES reporting in systematic reviews was consistently higher than in SCRD intervention studies. Specifically, 5 of 10 reviews (50.00%) reported ES estimates during 2001–2005, increasing to 10 of 17 reviews (58.82%) during 2006–2010, and peaking at 16 of 23 reviews (69.57%) during 2011–2015. ES reporting subsequently declined slightly, with 22 of 42 reviews (52.38%) during 2016–2020 and 16 of 32 reviews (50.00%) during 2021–2025 reporting ES estimates. Journal-level patterns indicated that Exceptional Children demonstrated the highest prevalence of ES reporting in systematic reviews, with 21 of 29 reviews (72.41%) reporting at least one ES estimate. In RASE, 37 of 73 reviews (50.68%) reported ES estimates, whereas in JSE, 11 of 22 reviews (50.00%) included ES estimates.

### 6.3. Types of Effect Size Metrics Reported in SCRD Intervention Studies

**Figure 4:**
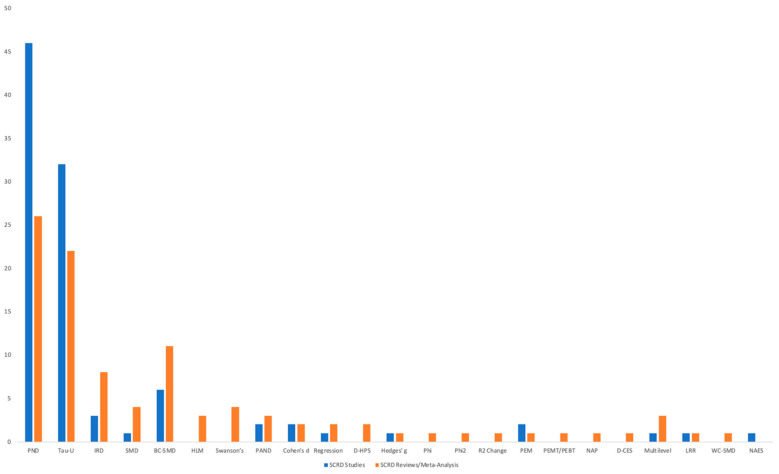
Frequency of Effect Size Methods Used in SCRD Studies and Reviews/Meta-Analysis. Note. PND = Percentage of Nonoverlapping Data; Tau-*U* = Kendall’s Tau with nonoverlap and trend correction; IRD = Improvement Rate Difference; SMD = Standardized Mean Difference; BC-SMD = Between-Case Standardized Mean Difference; WC-SMD = Within-Case Standardized Mean Difference; NAES = No-Assumptions Effect Size; PAND = Percentage of All Nonoverlapping Data; PEM = Percentage of Data Exceeding the Median; PEMT/PEBT = Percentage of Data Exceeding the Median Trend/Percentage of Data Exceeding the Baseline Trend; NAP = Nonoverlap of All Pairs; HLM = Hierarchical Linear Modeling; D-HPS = d-Hedges–Pustejovsky–Shadish; D-CES = Design-Comparable Effect Size; LRR = Log Response Ratio; SCRD = Single-Case Research Design.

Across the 82 SCRD intervention studies that reported at least one ES, most studies relied on a single ES metric, with 76 studies (91.57%) reporting one metric only, whereas 6 studies (8.43%) reported multiple ES metrics, ranging from two to five metrics per study. A total of 99 individual ES metrics were identified across these 82 studies (Table 3 and Figure 4). Overall, PND was the most frequently reported ES metric, accounting for 46 of the 99 metrics (46.46%). The second most frequently reported metric was Tau-*U*, which accounted for 32 metrics (32.32%). Together, PND and Tau-*U* comprised 78.78% of all ES metrics reported in SCRD intervention studies. All other metrics were used substantially less often, including BC-SMD (*n* = 6; 6.06%), IRD (*n* = 3; 3.03%), PAND (*n* = 2; 2.02%), Percentage of Data Exceeding the Median (PEM; *n* = 2; 2.02%), NAES (*n* = 1; 1.01%), Cohen’s d (*n* = 2; 2.02%), and Hedges’ g (*n* = 1; 1.01%). Finally, four additional methods were each reported once (*n* = 1; 1.01%): Log Response Ratio (LRR), multilevel models, Standardized Mean Difference (SMD), and regression-based ES. This indicates minimal adoption of these regression-based and standardized approaches in intervention-level SCRD studies.

Examination of temporal trends revealed substantial changes in ES metric usage across publication periods. During the 2001–2005 period, effect size estimates were provided in only two studies. Regarding the specific metrics employed, one study reported PND (*n* = 1; 50.00%) and the other reported Cohen’s d (*n* = 1; 50.00%). During 2006–2010, 12 ES metrics were reported, all of which were PND (100.00%), indicating exclusive reliance on overlap-based indices during this period. Between 2011–2015, the number of reported ES metrics increased to 23, although PND remained the dominant metric (*n* = 19; 82.61%). During this period, additional metrics began to appear, including Tau-*U* (*n* = 1; 4.35%), IRD (*n* = 2; 8.70%), and NAES (*n* = 1; 4.35%), reflecting the early adoption of alternative ES approaches.

During 2016–2020, 31 ES metrics were reported, representing a marked increase in methodological diversity. In this period, PND (*n* = 12; 38.71%) and Tau-*U* (*n* = 12; 38.71%) were reported at equal rates, followed by BC-SMD (*n* = 1; 3.23%), PAND (*n* = 2; 6.45%), PEM (*n* = 2; 6.45%), and IRD (*n* = 2; 6.45%). In the most recent period, 2021–2025, 31 ES metrics were again reported. Tau-*U* emerged as the most frequently reported metric (*n* = 19; 61.29%), whereas PND declined sharply to only 2 metrics (6.45%). Additional metrics reported during this period included BC-SMD (*n* = 5; 16.13%), IRD (*n* = 1; 3.23%), Cohen’s d (*n* = 1; 3.23%), Hedges’ g (*n* = 1; 3.23%), and several regression-based or model-based approaches (each *n* = 1; 3.23%). Overall, the distribution of ES metrics in SCRD intervention studies demonstrates a clear temporal shift from near-exclusive reliance on PND in earlier periods toward increasing adoption of Tau-U and limited use of model-based and standardized metrics in more recent years.

### 6.4. Types of Effect Size Metrics Reported in SCRD Systematic Reviews

Among the 69 SCRD systematic reviews that reported at least one ES estimate, a total of 100 individual ES metrics were identified (Table 4 and Figure 4), reflecting the use of both single and multiple ES approaches within reviews. Of these 69 reviews, 49 reviews (71.01%) reported a single ES metric, whereas 20 reviews (28.99%) reported multiple ES metrics, including 8 reviews (11.59%) that reported three or more distinct metrics and 2 reviews (2.90%) that reported five or more metrics.

Across all periods, the most frequently reported ES metric was PND, which accounted for 26 of the 100 reported metrics (26.00%). The second most frequently reported metric was Tau-*U*, with 22 metrics (22.00%), followed by between-case standardized mean difference (BC-SMD) with 11 metrics (11.00%). Other commonly reported metrics included IRD (*n* = 8; 8.00%), SMD (*n* = 4; 4.00%), hierarchical linear modeling approaches (HLM; *n* = 3; 3.00%), and Swanson’s procedure (*n* = 4; 4.00%). The remaining 22 metrics (22.00%) included PAND (*n* = 3; 3.00%), Multilevel models (*n* = 3; 3.00%), Cohen’s d (*n* = 2; 2.00%), Regression approaches (*n* = 2; 2.00%), and D-HPS (*n* = 2; 2.00%). Finally, ten other metrics were each reported once (*n* = 1; 1.00%), consisting of Hedges’ g, Phi, Phi², R² Change, PEM, PEMT/PEBT, NAP, D-CES, LRR, and WC-SMD.

Examination of temporal patterns revealed meaningful changes in ES metric usage across publication periods. During 2001–2005, among the systematic reviews that included effect size estimates, a total of five metric data points were identified. Specifically, PND was reported in two reviews (40.00%), Swanson’s procedure in two reviews (40.00%), and Cohen’s d in one review (20.00%). During 2006–2010, 10 ES metrics were reported, with PND remaining dominant (*n* = 6; 60.00%), followed by IRD (n = 4; 40.00%). Between 2011–2015, the number of reported ES metrics increased substantially to 25, reflecting greater methodological diversification. Although PND remained the most frequently reported metric (*n* = 10; 40.00%), additional metrics emerged more consistently, including Tau-*U* (*n* = 1; 4.00%), IRD (*n* = 3; 12.00%), SMD (*n* = 2; 8.00%), and BC-SMD (*n* = 5; 20.00%).

During 2016–2020, 37 ES metrics were reported, representing the highest number across all periods. In this period, Tau-*U* became the most frequently reported metric (*n* = 14; 37.84%), followed by PND (n = 7; 18.92%), BC-SMD (*n* = 5; 13.51%), and IRD (*n* = 3; 8.11%), alongside continued use of multilevel and regression-based approaches. In the most recent period, 2021–2025, 23 ES metrics were reported. Tau-*U* again emerged as the most frequently reported metric (*n* = 7; 30.43%), followed by BC-SMD (*n* = 6; 26.09%) and PND (*n* = 1; 4.35%). Compared to earlier periods, reliance on PND declined markedly, whereas the use of model-based and design-comparable ES metrics increased. Overall, these findings indicate that while PND remained the most frequently reported ES metric across all systematic reviews, there was a clear temporal shift toward greater methodological diversity, with increasing adoption of Tau-*U*, BC-SMD, and other statistically robust ES approaches in more recent publication periods.

## 7. Discussion

The primary objective of this study was to examine trends in effect size (ES) reporting practices in single-case research design (SCRD) studies published in three prominent special education journals, Journal of Special Education, Exceptional Children, and Remedial and Special Education, across a 25-year period (2001–2025). By systematically documenting the prevalence of SCRD intervention studies and SCRD systematic reviews, the frequency with which ES estimates were reported, and the types of ES metrics used, this study sought to clarify how SCRD researchers have responded to evolving expectations regarding EBPs and quantitative synthesis. Given the central role of ES estimates in meta-analysis and the identification of EBPs, the findings of this review provide timely insight into the methodological practices shaping the SCRD literature in special education.

Consistent with prior research, SCRD studies represented a meaningful proportion of published research in the three journals examined. Across the full review period, SCRD intervention studies accounted for approximately 10% of all published articles, with evidence of growth across publication periods. Although the most recent period (2021–2025) showed a noticeable decline in the proportion of SCRD intervention studies relative to 2016–2020, the broader pattern across two decades suggests sustained use of SCRD methodologies rather than a steady linear increase. This recent decline may reflect a temporary plateau or emerging shift in publication patterns, rather than a definitive reduction in the relevance or utility of SCRDs. These findings align with previous large-scale reviews indicating that SCRDs are among the most frequently employed methodologies in special education research (Alnahdi, 2015; Horner et al., 2005; King et al., 2024). The continued prominence of SCRD is likely attributable to the individualized nature of special education interventions and the practical and ethical challenges associated with group experimental designs when working with heterogeneous populations of students with disabilities (Lobo et al., 2017).

Importantly, the present findings indicate that increases in the publication of SCRD studies have been accompanied by important changes in ES reporting practices. Across journals, approximately 42% of SCRD intervention studies reported at least one ES estimate, with ES reporting increasing markedly over time—from fewer than 20% of studies in the early 2000s to nearly 72% of studies published between 2021 and 2025. This pattern represents a substantial shift in reporting norms and suggests growing alignment with recommendations to supplement visual analysis with quantitative indices of effect magnitude (Kratochwill et al., 2010; Maggin et al., 2021; Rakap, 2015).

In interpreting these trends, it is also important to consider the potential influence of major methodological guidance published during the review period. The 2005 SCRD standards (Horner et al., 2005) first established quality benchmarks, followed by the WWC Single-Case Design Technical Documentation (Kratochwill et al., 2010), one of the earliest formal recommendations to supplement visual analysis with effect size estimates, and subsequent pilot criteria (Kratochwill et al., 2013). WWC Standards Version 4.1 (2020) further prioritized design-comparable effect sizes for evidence reviews. These shifts coincided with evolving journal policies (Appelbaum et al., 2018). For example, Exceptional Children now requires ES reporting for SCRD studies. Federal evidence-based practice mandates under ESSA (2015) also reinforced the need for quantitative synthesis. Although this descriptive study cannot test causality, the temporal patterns increased ES reporting from 16.67% (2001–2005) to 71.88% (2021–2025), alongside Tau-U/BC-SMD adoption over PND appear consistent with these methodological standards, editorial expectations, and policy influences normalizing ES use in special education SCRD research.

Differences in ES reporting across Journal of Special Education (40.24%), Exceptional Children (47.83%), and Remedial and Special Education (40.91%) likely reflect varying editorial policies and their influence on research norms within the field. EC explicitly requires ES reporting for SCRD studies in its submission guidelines, which corresponds with its higher reporting rates, particularly in the most recent period (77.78% in 2021–2025). In contrast, JSE recommends ES reporting “when appropriate,” and RASE does not provide explicit guidance, aligning with their more moderate, though increasing, reporting trends. Beyond these differences, editorial policies within high-impact journals play a critical role in shaping broader research and reporting practices. Because JSE, EC, and RASE are widely regarded as leading outlets in special education, their author guidelines, reviewer expectations, and editorial decisions signal what is considered rigorous and acceptable research practice. As a result, researchers may adopt ES reporting not only to meet submission requirements for these journals but also to align their work with perceived field standards. Over time, such expectations can diffuse across other journals, graduate training programs, and research communities, contributing to the normalization of ES reporting in SCRD research. These leading journals therefore help establish methodological norms: publications with high impact factors (JSE: 2.2; EC: 4.3; RASE: 3.4) often set expectations for research design and reporting, as researchers tend to model their work on the standards of prestigious outlets (Appelbaum et al., 2018). The American Psychological Association and the American Educational Research Association guidelines mandating ES reporting (Wilkinson, 1999) further support this standardization, although their influence in special education has historically been less consistent. In this context, journal-level policies appear to amplify the impact of broader methodological guidance, such as WWC standards, by reinforcing expectations for quantitative reporting. This combined influence likely contributes to the increased adoption of metrics such as Tau-U and BC-SMD across the field.

Nevertheless, despite these improvements, a considerable proportion of SCRD intervention studies still did not report ES estimates, even in the most recent period. This finding echoes earlier reviews in related fields, including school psychology, where ES reporting in SCRD studies has historically remained below 50% (Radley et al., 2020). Taken together, these results suggest that although ES reporting has become more common, it has not yet reached the level of consistency observed in group-experimental research, where ES reporting is widely expected and often required (Cook et al., 2018; Thompson, 2007).

SCRD systematic reviews and meta-analyses demonstrated higher rates of ES reporting than intervention studies across all periods, with approximately 56% of reviews including at least one ES estimate. This pattern likely reflects the analytic demands of evidence synthesis, as ES estimates are essential for comparing effects across studies and drawing aggregate conclusions. However, even among systematic reviews, ES reporting plateaued in recent years at approximately 50%, suggesting that barriers to ES calculation and reporting persist even at the synthesis level.

Analysis of ES metric selection revealed additional insights into methodological evolution within the SCRD literature. Across both intervention studies and systematic reviews, PND was the most frequently reported ES metric in earlier publication periods. However, its dominance declined substantially over time, particularly in intervention studies, where its use decreased sharply in the most recent publication period. A similar, though less pronounced, pattern was observed in systematic reviews: although PND remained the most frequently reported metric overall, its use declined across later periods, coinciding with the increased adoption of Tau-*U*. In contrast, Tau-*U* demonstrated a clear rise in prominence over time, emerging as the most frequently reported metric in both SCRD intervention studies and systematic reviews during 2016–2020 and 2021–2025. This shift is notable given longstanding critiques of PND related to its sensitivity to baseline outliers and limited statistical properties (Parker et al., 2011; Wolery et al., 2010), along with growing recognition of Tau-*U*’s methodological advantages, particularly its ability to simultaneously account for baseline trend and nonoverlap.

The observed shift from PND dominance to Tau-*U* and BC-SMD reflects important differences in how these metrics are interpreted and the types of information they provide about intervention effects. Specifically, these metrics differ in (a) how they use data points, (b) whether they account for baseline trend, and (c) the scale on which effects are interpreted. The PND (Scruggs et al., 1987) calculates the proportion of intervention data points that exceed the highest baseline point, with values typically interpreted as 0 = no effect and above 70 indicating a strong effect. Although this interpretation is straightforward and easy to communicate, PND is based on a single extreme baseline value and does not account for trend or variability within phases. As a result, it may overestimate or underestimate intervention effects when baseline data are unstable or contain outliers (Parker et al., 2011; Rakap, 2015). In contrast, Tau-*U* (Parker et al., 2011) is interpreted as the proportion of nonoverlapping data across all pairwise comparisons between baseline and intervention phases, with values ranging from -1 to +1, where values closer to +1 indicate stronger positive intervention effects. Unlike PND, Tau-*U* incorporates all data points and can adjust for baseline trend, allowing researchers to distinguish true intervention effects from pre-existing improvement or decline. This results in a more stable and nuanced interpretation of intervention effectiveness, particularly for datasets with trend or variability, which are common in special education research (Yucesoy-Ozkan et al., 2020). The BC-SMD (Hedges et al., 2012) differs conceptually from both PND and Tau-*U* in that it is interpreted on a standardized mean difference scale comparable to Cohen’s d (e.g., 0.2 = small, 0.5 = medium, 0.8 = large). Rather than focusing on nonoverlap, BC-SMD quantifies the magnitude of change relative to variability across participants, enabling direct comparison of effects across studies and research designs. This makes BC-SMD particularly useful for meta-analysis and for integrating SCRD findings with group-experimental research, although it typically requires multiple cases and more complex computation (Pustejovsky, 2019; Pustejovsky et al., 2014). Taken together, these differences highlight a progression from simple descriptive indices (PND) toward metrics that provide more precise, stable, and comparable estimates of intervention effects. The increased use of Tau-*U* and BC-SMD reflects their stronger alignment with the needs of evidence synthesis, including sensitivity to data patterns, resistance to outliers, and compatibility with broader quantitative frameworks. These advantages likely contribute to their increased adoption in recent years while continuing to complement, rather than replace, visual analysis.

The increased use of Tau-*U*, BC-SMD, and other model-based or design-comparable ES metrics suggests that SCRD researchers are increasingly adopting methods that better accommodate the unique characteristics of single-case data and align more closely with contemporary standards for evidence synthesis. This trend is particularly evident in systematic reviews, which exhibited greater methodological diversity and more frequent use of multiple ES metrics. Such diversification is consistent with recommendations to report multiple ES estimates when appropriate, given the absence of consensus regarding a single optimal ES metric for SCRD (Vannest et al., 2018; Yucesoy-Ozkan et al., 2020).

Collectively, these findings reflect a field undergoing methodological transition. While early SCRD literature relied heavily on simple, overlap-based indices, more recent work has increasingly adopted statistically robust and design-sensitive ES approaches. Such robustness is characterized by greater sensitivity to key features of SCRD data, including serial dependence (autocorrelation), baseline trends, and data variability—features that are often overlooked by traditional nonoverlap indices. For instance, whereas PND has been criticized for its reliance on a single outlier data point and its susceptibility to ceiling effects (Parker et al., 2011), Tau-*U* provides a more stable and informative estimate by integrating nonoverlap with trend analysis (Parker et al., 2011). Similarly, BC-SMD represents a design-sensitive metric, as it explicitly corrects for small-sample bias and autocorrelation, thereby facilitating more meaningful comparisons with group-design effect sizes (Shadish et al., 2014). The increasing use of such design-comparable ES metrics is consistent with recommendations from the What Works Clearinghouse (WWC, 2020) and carries important implications for the integration of SCRD evidence into broader EBP syntheses alongside group-experimental research.

## 8. Limitations

Several limitations should be considered when interpreting the findings of this study. First, the review did not evaluate the methodological quality of the SCRD intervention studies or systematic reviews included in the analysis (Horner et al., 2005). As a result, it was not possible to determine whether higher-quality SCRD studies were more likely to report ES estimates or to use more sophisticated ES metrics. Importantly, increases in ES reporting over time likely reflect not only improvements in methodological rigor but also evolving reporting expectations and broader field-wide emphasis on quantitative synthesis alongside visual analysis. Advances in ES metrics, including nonoverlap indices (e.g., PND, PEM, and IRD; Parker et al., 2011) and standardized between-case effect sizes (Shadish et al., 2015), have also facilitated ES reporting. These trends have occurred alongside the adoption of widely cited SCRD standards (Horner et al., 2005; Kratochwill et al., 2013), which emphasize data sufficiency, replication, and internal validity, suggesting that increased ES reporting reflects both changing expectations and methodological developments rather than study quality alone. Future research that incorporates formal quality appraisal tools, such as the WWC standards, could provide important insights into the relationships among methodological rigor, reporting practices, and ES trends.

Second, the scope of the review was limited to three high-impact special education journals. Although these journals play a central role in shaping research standards within the field, ES reporting practices in other outlets such as behavior analytic journals may differ. Consequently, the generalizability of the findings beyond these journals is limited. Expanding future reviews to include a broader range of publication venues would yield a more comprehensive understanding of ES reporting practices across disciplines.

Third, the review focused exclusively on published studies, raising the possibility of publication bias. SCRD studies with weaker or null intervention effects may be less likely to be published and, correspondingly, less likely to report ES estimates. Additionally, unpublished studies and gray literature may follow different reporting conventions. As such, the observed prevalence of ES reporting may overestimate or underestimate actual practices within the broader SCRD research community.

Finally, this study did not examine the appropriateness of the selected ES metrics relative to the characteristics of the dependent variables or data structures used in individual studies. Although this was beyond the scope of the present review, future research examining the alignment between ES metric selection and data characteristics would provide valuable guidance for improving ES reporting practices in SCRD.

## 9. Implications for Research and Practice

The findings of this review have several important implications for researchers, journal editors, and the broader special education community. First, continued efforts are needed to normalize ES reporting in SCRD intervention studies, particularly given the central role of ES estimates in identifying EBPs. Journal editors may consider adopting clearer guidelines or expectations regarding ES reporting in SCRD manuscripts, similar to those long established for group-experimental research. Second, the observed shift toward Tau-*U*, BC-SMD, and other design-sensitive metrics highlights the importance of methodological training for SCRD researchers. Graduate programs and professional development initiatives should emphasize not only how to compute ES estimates but also how to select metrics that align with specific data characteristics and research purposes.

Third, greater transparency in data reporting would substantially enhance the utility of SCRD research. Although raw outcome data in SCRD studies are typically presented graphically within published articles, encouraging authors to provide the numerical values underlying these graphs as supplementary materials would facilitate secondary analyses, meta-analyses, and replication efforts, thereby strengthening the evidence base for EBPs in special education. Finally, the increasing use of design-comparable ES metrics offers a promising pathway for integrating SCRD findings with group-experimental research. Such integration is critical for advancing inclusive and comprehensive evidence syntheses that reflect the diversity of research designs used in special education.

## 10. Conclusions

This study provides a comprehensive examination of ES reporting trends in SCRD research published across three major special education journals over a 25-year period. The findings indicate that SCRD studies remain a prominent methodological approach in special education and that ES reporting practices have improved over time, particularly in more recent publication periods. At the same time, ES reporting remains inconsistent, with many SCRD intervention studies still omitting ES estimates, underscoring the need for continued methodological refinement and clearer reporting standards. Variability in reporting frequency and metric selection further highlights ongoing challenges in establishing ES reporting as a routine practice within SCRD research. The observed shift away from exclusive reliance on PND toward greater use of Tau-*U*, BC-SMD, and other advanced ES methods reflects meaningful progress in the field. However, this progress has not yet reached the level of consistency commonly expected in group-experimental research, suggesting that additional efforts are needed to normalize ES reporting across SCRD studies. Future research reports would benefit from adopting reporting practices that integrate traditional visual analysis with complementary quantitative summaries of intervention effects. Visual inspection of graphed data should remain the primary method for establishing functional relations in SCRD studies; however, reporting effect size estimates alongside visual analysis can improve transparency, facilitate comparison across studies, and support the synthesis of findings in systematic reviews and meta-analyses. Researchers are therefore encouraged to report effect size metrics that align with the characteristics of single-case data while clearly describing how these metrics complement visual interpretations of intervention effects. Opportunities therefore remain to further strengthen ES reporting practices, enhance methodological transparency, and support the integration of SCRD findings into EBP frameworks. Establishing clearer expectations for combining visual analysis with appropriate effect size reporting may help promote greater consistency in reporting practices across SCRD studies. Continued attention to these issues will be essential for advancing the quality, interpretability, and impact of SCRD research in special education.

## Figures and Tables

**Table 1:** Number and Percentage of SCRD Articles Published and Reported Effect Size.

Publication Year	Total Number of			Percentage of	
	Articles Published	SCRD Articles Published	SCRD Articles Reported ES	SCRD Articles Published	SCRD Articles Reported ES
Journal of Special Education					
2001–2005	96	6	0	6.25	0.00
2006–2010	89	14	5	15.73	35.71
2011–2015	102	22	9	21.57	40.91
2016–2020	107	25	10	23.36	40.00
2021–2025	106	15	9	14.15	60.00
Total	500	82	33	16.4	40.24
Exceptional Children					
2001–2005	138	4	1	2.90	25.00
2006–2010	125	10	6	8.00	60.00
2011–2015	138	14	4	10.14	28.57
2016–2020	122	9	4	7.38	44.44
2021–2025	112	9	7	8.04	77.78
Total	635	46	22	7.24	47.83
Remedial and Special Education					
2001–2005	172	2	1	1.16	50.00
2006–2010	163	12	1	7.36	8.33
2011–2015	182	24	10	13.19	41.67
2016–2020	152	20	8	13.16	40.00
2021–2025	165	8	7	4.85	87.50
Total	834	66	27	7.91	40.91
Combined					
2001–2005	406	12	2	2.96	16.67
2006–2010	377	36	12	9.55	33.33
2011–2015	422	60	23	14.22	38.33
2016–2020	381	54	22	14.17	40.74
2021–2025	383	32	23	8.36	71.88
Total	1969	194	82	9.85	42.27

Note. SCRD = Single-Case Research Design; ES = Effect Size.

**Table 2:** Number and Percentage of Systematic Reviews Published and Reported Effect Size.

Publication Year	Total Number of			Percentage of	
	Articles Published	SCRD Reviews/Meta-Analysis Published	SCRD Reviews/Meta-Analysis Reported ES	SCRD Reviews/Meta-Analysis Published	SCRD Reviews/Meta-Analysis Reported ES
Journal of Special Education					
2001–2005	96	0	0	0	0
2006–2010	89	1	0	1.12	0
2011–2015	102	4	3	3.92	75.00
2016–2020	107	7	4	6.54	57.14
2021–2025	106	10	4	9.43	40.00
Total	500	22	11	4.40	50.00
Exceptional Children					
2001–2005	138	3	2	2.17	66.67
2006–2010	125	9	4	7.20	44.44
2011–2015	138	5	5	3.62	100.00
2016–2020	122	7	6	5.74	85.71
2021–2025	112	5	4	4.46	80.00
Total	635	29	21	4.57	72.41
Remedial and Special Education					
2001–2005	172	7	3	4.07	42.86
2006–2010	163	7	6	4.29	85.71
2011–2015	182	14	8	7.69	57.14
2016–2020	152	28	12	18.42	42.86
2021–2025	165	17	8	10.30	47.06
Total	834	73	37	8.75	50.68
Combined					
2001–2005	406	10	5	2.46	50.00
2006–2010	377	17	10	4.51	58.82
2011–2015	422	23	16	5.45	69.57
2016–2020	381	42	22	11.02	52.38
2021–2025	383	32	16	8.36	50.00
Total	1969	124	69	6.30	55.65

Note. SCRD = Single-Case Research Design; ES = Effect Size.

**Table 3:** Effect Size Methods Used in SCRD Studies Across Years.

Method	2001–2005	2006–2010	2011–2015	2016–2020	2021–2025	Total
PND	1 (50)	12 (100)	19 (83)	12 (39)	2 (7)	46 (46)
Tau-*U*	-	-	1 (4)	12 (39)	19 (61)	32 (31)
BC-SMD	-	-	-	1(3)	5 (16)	6 (6)
IRD	-	-	2 (9)	-	1 (3)	3 (3)
PAND	-	-	-	2 (7)	-	2 (2)
PEM	-	-	-	2 (7)	-	2 (2)
Cohen’s d	1 (50)	-	-	1(3)	-	2 (2)
NAES	-	-	1 (4)	-	-	1 (1)
Hedges’ g	-	-	-	1 (3)	-	1 (1)
LRR	-	-	-	-	1 (3)	1 (1)
Multi-level	-	-	-	-	1 (3)	1 (1)
SMD	-	-	-	-	1 (3)	1 (1)
Regression	-	-	-	-	1 (3)	1 (1)
Total	2	12	23	31	31	99

Note. Values represent frequencies (percentages). PND = Percentage of Nonoverlapping Data; Tau-*U* = Kendall’s Tau with nonoverlap and trend correction; BC-SMD = Between-Case Standardized Mean Difference; IRD = Improvement Rate Difference; PAND = Percentage of All Nonoverlapping Data; PEM = Percentage of Data Exceeding the Median; NAES = No-Assumptions Effect Size; LRR = Log Response Ratio; SMD = Standardized Mean Difference; SCRD = Single-Case Research Design.

**Table 4:** Effect Size Methods Reported in SCRD Systematic Reviews Across Years.

Method	2001–2005	2006–2010	2011–2015	2016–2020	2021–2025	Total
PND	2 (40)	6 (60)	10 (40)	7 (19)	1 (4)	26 (26)
Tau-*U*	-	-	1 (4)	14 (38)	7 (30)	22 (22)
IRD	-	-	4 (10)	3 (8)	1 (4)	8 (7)
SMD	-	-	2 (8)	2 (5)	-	4 (4)
BC-SMD	-	-	-	5 (14)	6 (26)	11 (11)
HLM	-	-	2 (8)	-	1 (4)	3 (3)
Swanson’s	2 (40)	2 (20)	-	-	-	4 (4)
PAND	-	-	2 (8)	1 (3)	-	3 (3)
Multilevel	-	-	-	-	3 (13)	3 (3)
Cohen’s d	1 (20)	1 (10)	-	-	-	2 (2)
Regression	-	-	1 (4)	1 (3)	-	2 (2)
D-HPS	-	-	-	1 (3)	1 (4)	2 (2)
Hedges’ g	-	1 (10)	-	-	-	1 (1)
Phi	-	-	1 (4)	-	-	1 (1)
Phi^2^	-	-	1 (4)	-	-	1 (1)
R^2^ Change	-	-	1 (4)	-	-	1 (1)
PEM	-	-	-	1 (3)	-	1 (1)
PEMT/PEBT	-	-	-	1 (3)	-	1 (1)
NAP	-	-	-	1 (3)	-	1 (1)
D-CES	-	-	-	-	1 (4)	1 (1)
LRR	-	-	-	-	1 (4)	1 (1)
WC-SMD	-	-	-	-	1 (4)	1 (1)
Total	5	10	25	37	23	100

Note. Values represent frequencies (percentages). PND = Percentage of Nonoverlapping Data; Tau-*U* = Kendall’s Tau with nonoverlap and trend correction; IRD = Improvement Rate Difference; SMD = Standardized Mean Difference; BC-SMD = Between-Case Standardized Mean Difference; HLM = Hierarchical Linear Modeling; PAND = Percentage of All Nonoverlapping Data; PEM = Percentage of Data Exceeding the Median; PEMT/PEBT = Percentage of Data Exceeding the Median Trend/Percentage of Data Exceeding the Baseline Trend; NAP = Nonoverlap of All Pairs; D-HPS = d-Hedges–Pustejovsky–Shadish; D-CES = Design-Comparable Effect Size; LRR = Log Response Ratio; WC-SMD = Within-Case Standardized Mean Difference; SCRD = Single-Case Research Design.

## Data Availability

Data available on request from the first author.

## References

[B1-behavsci-16-00507] Alnahdi G. H. (2015). Single-subject designs in special education: Advantages and limitations. Journal of Research in Special Educational Needs.

[B2-behavsci-16-00507] Appelbaum M., Cooper H., Kline R. B., Mayo-Wilson E., Nezu A. M., Rao S. M. (2018). Journal article reporting standards for quantitative research in psychology: The APA Publications and Communications Board task force report. American Psychologist.

[B3-behavsci-16-00507] Balıkcı Ş., Kalkan S., Rakap S., Akemoğlu Y. (2022). Investigation of five nonoverlap methods to calculate effect sizes in single-case research. Journal of Education for Life.

[B4-behavsci-16-00507] Beretvas S. N., Chung H. (2008). A review of meta-analyses of single-subject experimental designs: Methodological issues and practice. Evidence-Based Communication Assessment and Intervention.

[B5-behavsci-16-00507] Brossart D. F., Parker R. I., Olson E. A., Mahadevan L. (2006). The relationship between visual analysis and five statistical analyses in a simple AB single-case research design. Behavior Modification.

[B6-behavsci-16-00507] Cook B. G., Buysse V., Klingner J., Landrum T. J., McWilliam R. A., Tankersley M., Test D. W. (2015). CEC’s standards for classifying the evidence base of practices in special education. Remedial and Special Education.

[B7-behavsci-16-00507] Cook B. G., Collins L. W., Cook S. C., Cook L. (2020). Evidence-based reviews: How evidence-based practices are systematically identified. Learning Disabilities Research & Practice.

[B8-behavsci-16-00507] Cook B. G., Cook L., Therrien W. J. (2018). Group-difference effect sizes: Gauging the practical importance of findings from group-experimental research. Learning Disabilities Research & Practice.

[B9-behavsci-16-00507] Cook B. G., Cook S. C. (2011). Unraveling evidence-based practices in special education. Journal of Special Education.

[B10-behavsci-16-00507] Cook B. G., Odom S. L. (2013). Evidence-based practices and implementation science in special education. Exceptional Children.

[B11-behavsci-16-00507] Cook B. G., Smith G. J., Tankersley M., Harris K. R., Graham S., Urdan T. (2011). Evidence-based practices in education. APA educational psychology handbook.

[B12-behavsci-16-00507] Durlak J. A. (2009). How to select, calculate, and interpret effect sizes. Journal of Pediatric Psychology.

[B13-behavsci-16-00507] ESSA (2015). Every student succeeds act, 20 U.S.C. § 6301.

[B14-behavsci-16-00507] Farmer J. L., Owens C. M., Ferron J. M., Allsopp D. H. (2010). A methodological review of single-case meta-analyses *[Paper presentation]*. The American Educational Research Association.

[B15-behavsci-16-00507] Hedges L. V., Pustejovsky J. E., Shadish W. R. (2012). A standardized mean difference effect size for single case designs. Research Synthesis Methods.

[B16-behavsci-16-00507] Horner R. H., Carr E. G., Halle J., McGee G., Odom S., Wolery M. (2005). The use of single-subject research to identify evidence-based practice in special education. Exceptional Children.

[B17-behavsci-16-00507] Jamshidi L., Heyvaert M., Declercq L., Fernández-Castilla B., Ferron J. M., Moeyaert M., Beretvas N., Onghena P., Van den Noortgate W. (2022). A systematic review of single-case experimental design meta-analyses: Characteristics of study designs, data, and analyses. Evidence Based Communication Assessment and Intervention.

[B18-behavsci-16-00507] King S. A., Nylen B., Enders O., Wang L., Opeoluwa O. (2024). Examining the impact of design-comparable effect size on the analysis of single-case design in special education. School Psychology.

[B19-behavsci-16-00507] Kraft M. A. (2020). Interpreting effect sizes of education interventions. Educational Researcher.

[B20-behavsci-16-00507] Kratochwill T. R., Hitchcock J., Horner R. H., Levin J. R., Odom S. L., Rindskopf D. M., Shadish W. R. (2010). Single-case designs technical documentation.

[B21-behavsci-16-00507] Kratochwill T. R., Hitchcock J. H., Horner R. H., Levin J. R., Odom S. L., Rindskopf D. M., Shadish W. R. (2013). Single-case intervention research design standards. Remedial and Special Education.

[B22-behavsci-16-00507] Kratochwill T. R., Horner R. H., Levin J. R., Machalicek W., Ferron J., Johnson A. (2021). Single-case design standards: An update and proposed upgrades. Journal of School Psychology.

[B23-behavsci-16-00507] Ledford J. R., Lambert J. M., Pustejovsky J. E., Zimmerman K. N., Hollins N., Barton E. E. (2023). Single-case-design research in special education: Next-generation guidelines and considerations. Exceptional Children.

[B24-behavsci-16-00507] Ledford J. R., Lane J. D., Gast D. L., Ledord J. R., Gast D. L. (2018a). Dependent variables, measurement, and reliability. Single case research methodology: Applications in special education and behavioral sciences.

[B25-behavsci-16-00507] Ledford J. R., Lane J. D., Severini K. E. (2018b). Systematic use of visual analysis for assessing outcomes in single case design studies. Brain Impairment.

[B26-behavsci-16-00507] Lobo M. A., Moeyaert M., Cunha A. B., Babik I. (2017). Single-case design, analysis, and quality assessment for intervention research. Journal of Neurologic Physical Therapy: JNPT.

[B27-behavsci-16-00507] Maggin D. M., Barton E., Reichow B., Lane K., Shogren K. A. (2021). Commentary on the what works clearinghouse standards and procedures handbook (v. 4.1) for the review of single-case research. Remedial and Special Education.

[B28-behavsci-16-00507] Maggin D. M., Briesch A. M., Chafouleas S. M., Ferguson T. D., Clark C. (2014). A comparison of rubrics for identifying empirically supported practices with single-case research. Journal of Behavioral Education.

[B29-behavsci-16-00507] Maggin D. M., O’Keeffe B. V., Johnson A. H. (2011). A quantitative synthesis of methodology in the meta-analysis of single-subject research for students with disabilities: 1985–2009. Exceptionality: A Special Education Journal.

[B30-behavsci-16-00507] Mitchell R. E., Karchmer M. A. (2004). Chasing the mythical ten percent: Parental hearing status of deaf and hard of hearing students in the United States. Sign Language Studies.

[B31-behavsci-16-00507] Moeyaert M., Zimmerman K. N., Ledford J. R., Ledford J. R., Gast D. L. (2018). Synthesis and meta-analysis of single case research. Single case research methodology: Applications in special education and behavior sciences.

[B32-behavsci-16-00507] Parker R. I., Vannest K. J., Davis J. L., Kratochwill T. R., Levin J. R. (2014). Non-overlap analysis for single-case research. Single-case intervention research: Methodological and statistical advances.

[B33-behavsci-16-00507] Parker R. I., Vannest K. J., Davis J. L., Sauber S. B. (2011). Combining nonoverlap and trend for single-case research: Tau-U. Behavior Therapy.

[B34-behavsci-16-00507] Peng C. Y. J., Chen L. T., Chiang H. M., Chiang Y. C. (2013). The impact of APA and AERA guidelines on effect size reporting. Educational Psychology Review.

[B35-behavsci-16-00507] Pustejovsky J. E. (2019). Procedural sensitivities of effect sizes for single-case designs with directly observed behavioral outcome measures. Psychological Methods.

[B36-behavsci-16-00507] Pustejovsky J. E., Ferron J. M., Kaufmann J. M., Hallahan D. P., Pullen P. C. (2017). Research synthesis and meta-analysis of single-case designs. Handbook of special education.

[B37-behavsci-16-00507] Pustejovsky J. E., Hedges L. V., Shadish W. R. (2014). Design-comparable effect sizes in multiple baseline designs: A general modeling framework. Journal of Educational and Behavioral Statistics.

[B38-behavsci-16-00507] Radley K. C., Dart E. H., Fischer A. J., Collins T. A. (2020). Publication trends for single-case methodology in school psychology: A systematic review. Psychology in the Schools.

[B39-behavsci-16-00507] Rakap S. (2015). Effect sizes as result interpretation aides in single-subject experimental research: Description and application of four nonoverlap methods. British Journal of Special Education.

[B40-behavsci-16-00507] Rakap S., Snyder P., Pasia C. (2014). Comparison of nonoverlap methods for identifying treatment effect in single-subject experimental research. Behavioral Disorders.

[B41-behavsci-16-00507] Rakap S., Yucesoy-Ozkan S., Kalkan S. (2020). Effect size calculations in single subject experimental research design: A review of nonoverlap methods. Turkish Journal of Psychology.

[B42-behavsci-16-00507] Rodgers W., Lewis T., O’Neill R., Vannest K. (2018). Policy and position statement on single case research and experimental designs.

[B43-behavsci-16-00507] Schäfer T., Schwarz M. A. (2019). The meaningfulness of effect sizes in psychological research: Differences between sub-disciplines and the impact of potential biases. Frontiers in Psychology.

[B44-behavsci-16-00507] Scruggs T. E., Mastropieri M. A., Casto G. (1987). The quantitative synthesis of single subject research: Methodology and validation. Remedial and Special Education.

[B45-behavsci-16-00507] Shadish W. R., Hedges L. V., Horner R. H., Odom S. L. (2015). The role of between-case effect size in conducting, interpreting, and summarizing single-case research (NCER 2015-002).

[B46-behavsci-16-00507] Shadish W. R., Hedges L. V., Pustejovsky J. E. (2014). Analysis and meta-analysis of single-case designs with a standardized mean difference statistic: A primer and applications. Journal of School Psychology.

[B47-behavsci-16-00507] Thompson B. (2007). Effect sizes, confidence intervals, and confidence intervals for effect sizes. Psychology in the Schools.

[B48-behavsci-16-00507] Vannest K. J., Peltier C., Haas A. (2018). Results reporting in single case experiments and single case meta-analysis. Research in Developmental Disabilities.

[B49-behavsci-16-00507] What Works Clearinghouse (2020). What works clearinghouse standards handbook, version 4.1.

[B50-behavsci-16-00507] Wilkinson L. (1999). Statistical methods in psychology journals: Guidelines and explanations. American Psychologist.

[B51-behavsci-16-00507] Wolery M. (2013). A commentary: Single-case design technical standards. Remedial and Special Education.

[B52-behavsci-16-00507] Wolery M., Busick M., Reichow B., Barton E. E. (2010). Comparison of overlap methods for quantitatively synthesizing single-subject data. The Journal of Special Education.

[B53-behavsci-16-00507] Wolery M., Dunlap G., Ledford J. R. (2011). Single-case experimental methods: Suggestions for reporting. Journal of Early Intervention.

[B54-behavsci-16-00507] Yucesoy-Ozkan S., Rakap S., Gulboy E. (2020). Evaluation of treatment effect estimates in single-case experimental research: Comparison of twelve overlap methods and visual analysis. British Journal of Special Education.

